# Polyphenolic Profile and Biological Activities in HT29 Intestinal Epithelial Cells of *Feijoa sellowiana* Fruit Extract

**DOI:** 10.3390/ijms26167851

**Published:** 2025-08-14

**Authors:** Paola Faraoni, Margherita Campo, Alessio Gnerucci, Pamela Vignolini, Francesco Ranaldi, Teresa Iantomasi, Lorenzo Bini, Massimo Gori, Edgardo Giordani, Roberto Natale, Stefania Nin, Roberto Carossino, Stefano Biricolti

**Affiliations:** 1 Department of Experimental and Clinic Biomedical Sciences “Mario Serio”, University of Florence, Viale Pieraccini 6, 50139 Florence, FI, Italy; paola.faraoni@unifi.it (P.F.); teresa.iantomasi@unifi.it (T.I.); roberto.carossino@unifi.it (R.C.); 2 Department of Statistic Informatic Application “G. Parenti” (DiSIA), PHYTOLAB Laboratory, University of Florence, Via Ugo Schiff 6, 50019 Sesto Fiorentino, FI, Italy; margherita.campo@unifi.it (M.C.); pamela.vignolini@unifi.it (P.V.); 3 Department of Physics and Astronomy, University of Florence, Via Sansone 1, 50019 Sesto Fiorentino, FI, Italy; 4 Department of Agriculture, Food, Environment and Forestry (DAGRI), University of Florence, Viale delle Idee 30, 50019 Sesto Fiorentino, FI, Italy; lorenzo.bini@unifi.it (L.B.); massimo.gori@unifi.it (M.G.); edgardo.giordani@unifi.it (E.G.); roberto.natale@unifi.it (R.N.); stefano.biricolti@unifi.it (S.B.); 5 Interdepartmental Service Center for Agricultural, Chemical and Industrial Biotechnology (CIBIACI), University of Florence, Via Romana 21, 50125 Florence, FI, Italy; 6 Council for Agricultural Research and Economics, Research Centre for Vegetable and Ornamental Crops, 51017 Pescia, PT, Italy; stefania.nin@crea.gov.it

**Keywords:** *Feijoa sellowiana*, HT29 cells, polyphenols, HPLC-DAD-MS analysis, oxidative stress, enzymatic activity, antioxidant properties

## Abstract

Oxidative and inflammatory stresses contribute to the development of many intestinal pathologies. This study characterized the polyphenolic profile and biological activity of a hydroalcoholic extract obtained from the fruit pulp of *Feijoa sellowiana* on HT29 intestinal epithelial cells subjected to oxidative (H_2_O_2_) and inflammatory (cytokines) stress. HPLC-DAD-MS analysis revealed an interesting phenolic composition, rich in hydrolyzable tannins (HHDP-glucose, pedunculagin and other ellagic acid derivatives) and condensed tannins (procyanidin dimers), with a total polyphenol content of 8.07 mg/g GAE. The extract was non-cytotoxic up to 160 µg/mL and exerted a protective effect against the cytokine-induced reduction in cell viability. In vitro assays confirmed its strong antioxidant and scavenging capacity. The scratch assay suggested enhanced cell migration. The extract modulated the activity of key metabolic enzymes restoring glucose-6-phosphate dehydrogenase and enolase activity, while supporting glycolytic flux through pyruvate kinase and lactate dehydrogenase. PCA and Pearson correlation analyses confirmed a treatment-dependent modulation of the metabolic and redox profile, suggesting a regulatory role beyond a mere scavenging effect. These findings highlight the nutraceutical potential of feijoa polyphenols, not only as direct antioxidants but also as modulators of cellular metabolism and redox homeostasis, supporting their application in gastrointestinal disorders with oxidative or inflammatory components.

## 1. Introduction

Climate change and different cultivation methods have led to the possibility of cultivating, even extensively, different plant varieties at unusual latitudes. Indeed, it is not uncommon to observe, for example, the cultivation of plants of tropical origin at latitudes defined as temperate, which are now able not only to flower but also to bear fruit [1,2,3]. These changes in cultivation are then reflected in the market with the consequent increased availability of fruit and vegetables previously considered exotic [4,5,6,7].

Among the crops that are also becoming increasingly popular in Europe is *Feijoa sellowiana* (O. Berg), a plant native to South America, whose edible fruits, as well as the peel and leaves, are rich in bioactive compounds, such as polyphenols, vitamins C and E, folic acid and iodine [8,9,10,11]. However, due to the difficulties of agronomical practices (e.g., clonal propagation) [12,13] and a short shelf–life of its fruit, feijoa diffusion and consumption have been limited, and it is still considered a minor fruit [14,15,16].

In any case, the increased cultivation and consumption of this fruit inevitably lead to an increase in production and consumption waste that can, given its richness in biologically active compounds, be valorised in circular economy circuits [17,18]. Extracts obtained from feijoa fruit, peels and leaves have shown antioxidant, anti-inflammatory [8,10,19], anti-microbial [11,20,21,22] and even anti-cancer activity [23,24]. For instance, feijoa extracts have been found to inhibit the production of superoxide anion in human neutrophils [19], to reduce lipid peroxidation in in vivo tests on rats [10] and to reduce the inflammatory state induced by Toll-Like Receptor 2 and 4 in human embryonic kidney cells and in an intestinal cell line (HCT15) [25,26]. The antitumour role of feijoa extracts was tested on cell lines of different origin, from haematological tumours and solid tumours, such as prostate and head and neck cancers, and seems to be related to the content of proanthocyanidins and flavones [23,24,27]. The anti-microbial action observed against both Gram-positive bacteria such as *S. aureus* and Gram-negative bacteria such as *E. coli*, as well as against fungi such as *C. albicans* seems to be related to polyphenols, specifically ellagitannins and synergic acid [11,20,21,22,28]. Extracts obtained from leaves showed antidiabetic action as they were able to inhibit enzymes such as alpha glucosidase and alpha amylase in rats. The inhibitory action of enzyme activities by feijoa leaves extracts was also observed against acetylcholinesterase and lipase [20,29].

The presence of biologically active molecules in feijoa extracts may therefore lead to the idea of new feijoa-based functional foods in order to benefit from its nutraceutical properties.

With this in mind, it is therefore crucial to produce extracts from feijoa fruits and wastes in order to maximise and refine the recovery of bioactive molecules, whose health-promoting properties are increasingly being studied. The development and optimisation of extraction protocols and the characterisation of the chemical and biological effects of these extracts have consequently become an important step in the process of developing new functional foods.

The present work is part of a wide-ranging research study focused on *Feijoa sellowiana*, involving the study of some agronomic, genetic [30], in vitro culture and nutraceutical aspects.

In this study, a quali-quantitative evaluation of the polyphenol content in a hydroalcoholic extract of feijoa fruit pulp was performed by in vitro assay with Folin–Ciocalteu reagent and HPLC-DAD-MS analysis; then, the antioxidant and cytoprotective properties and the ability to influence the activity of enzymes involved in the metabolism were investigated in a cell line derived from an adenocarcinoma of the rectum, HT29, which was recognised as a model for testing these properties of foods and dietary supplements [31,32]. Therefore, the choice of these cells is mainly due to the fact that many intestinal diseases have at their base an inflammatory state that causes oxidative stress [33]. Consequently, the introduction into the diet of bioactive compounds capable of counteracting such stress can be a valid preventative as well as therapeutic approach.

## 2. Results and Discussion

The objective of this study was to evaluate the effect of the hydroalcoholic extract of *Feijoa sellowiana* (feijoa) on cell viability, reactive oxygen species (ROS) production, enzymatic activity of key metabolic markers and multivariate analysis of enzymatic responses in HT29 intestinal epithelial cells subjected to oxidative (hydrogen peroxide, H_2_O_2_) and inflammatory (pro-inflammatory cytokines) stress conditions.

### 2.1. Phenolic Compounds in Feijoa Fruit Pulp Extract

Total phenolic content was evaluated by in vitro spectrophotometric assay with Folin–Ciocalteu reagent. This test is widely used for the screening of total phenols and polyphenols in natural extracts from plant material, foods and drinks, and the obtained results showed a direct correlation with the antioxidant activity evaluated with other antioxidant assays such as ORAC, ABTS and DPPH [34]. The calibration was performed using gallic acid as an external standard, and the results were expressed as the concentration of total phenolics in gallic acid equivalents (GAEs). The determined concentration of total phenols and polyphenols was 8.07 ± 1.01 mg/g GAEs with respect to the weight of freeze-dried pulp (1.37 mg/g GAEs with respect to the weight of fresh pulp with a water content of 83%).

**Figure 1:**
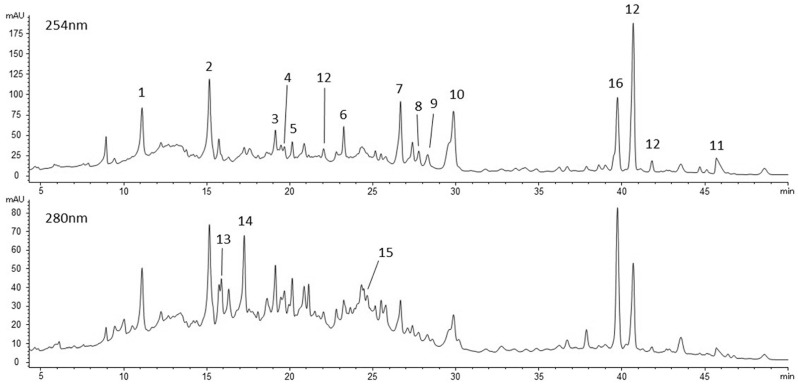
Chromatographic profile of feijoa extract, at 254 nm and 280 nm. 1. Pedunculagin isomer I; 2. pedunculagin isomer II; 3. HHDP-glucose; 4. flavonoid derivative; 5. casuarictin/potentillin; 6. ellagic acid hexoside I; 7. ellagic acid arabinoside I; 8. ellagic acid arabinoside II; 9. ellagic acid hexoside II; 10. ellagic acid derivative *m*/*z* 491; 11. ellagic acid derivative *m*/*z* 423; 12. ellagic acid derivatives calibrated as ellagic acid; 13. procyanidin dimer I; 14. procyanidin dimer II; 15. procyanidin dimer III; 16. unknown compound calibrated as ellagic acid.

In the literature, data are available about the quali-quantitative characterization by chromatographic techniques of the polyphenol content of various parts of feijoa plant, including fruit pulp, showing the complexity of its composition. The reported data generally show the presence of flavonoids, derivatives of ellagic acid or hexahydroxydiphenoyl-glucose (HHDP-glucose) and other phenolic and polyphenolic derivatives such as procyanidins, flavan-3-ols, derivatives of hydroxycinnamic, gallic, vanillic, syringic and quinic acids. The results relating to the presence of specific subclasses, individual derivatives and their quantitative ratios are variable [35,36,37,38]. In the present study, the HPLC-DAD-MS profile of the freeze-dried pulp extract confirmed the complex polyphenol composition that made the chromatographic separation of the mixture difficult. In Figure 1, the HPLC-DAD-MS chromatographic profile of feijoa fruit extract is shown, acquired at 254 nm and 280 nm, and Table 1 shows the results of the quali-quantitative characterization; the detected compounds are progressively numbered. The reported compounds showed UV-Vis absorption maxima mainly around 254 nm and 280 nm and molecular weights ranging from 302 Da (ellagic acid) up to 936 Da (casuarictin/potentillin). Except for a flavonoid derivative detected only in traces (4) and one unknown compound (16), the other compounds belong to the polyphenolic subclasses of hydrolysable and condensed tannins. The identified hydrolysable tannins were two isomers of pedunculagin (1 and 2), HHDP-glucose (3), casuarictin/potentillin (galloylated form of pedunculagin, 5), two ellagic acid hexosides (6 and 9) and two isomers of ellagic acid penthoside (7 and 8). These latter were tentatively identified as ellagic acid arabinoside isomers according to Aoyama et al. [27]. Compounds 10 and 11 showed UV-Vis absorption profiles attributable to ellagic acid derivatives, but the identification was not possible according to their molecular weights; they were reported as ellagic acid derivatives calibrated in ellagic acid and corrected for their molecular weights. Three more compounds were detected and identified as generic ellagic derivatives according to their UV-Vis absorption profiles; in this case, molecular weights were not clearly defined due to co-elution or poor ionization effect, so these compounds calibrated as ellagic acid and their amounts were summed (12). The chromatographic peak n. 16 is due to the co-elution of several compounds, the main one of which appears to have maximum UV-Vis absorbance around 270 nm and a molecular weight not exactly defined due to the presence of co-eluted compounds. This unknown compound was calibrated as ellagic acid, but more studies are necessary to resolve its chromatographic peak and for its correct identification. Condensed tannins are present as three different dimeric B-type procyanidins (13–15).

From a qualitative point of view, the obtained results agree with the data available in the literature [27,39,40]. These results indicate the presence of an interesting content and a large variety of polyphenols belonging almost entirely to the subclasses of hydrolyzable and condensed tannins, characterized by numerous biological properties including antioxidant activity. Further studies are necessary for a more in-depth quali-quantitative characterization of the polyphenolic content of feijoa fruit pulp.

### 2.2. Scavenging Activity and Total Antioxidant Capacity

The DPPH (2,2-diphenyl-1-picrylhydrazy) test showed that the feijoa extract (concentration of polyphenolic compounds 20.88 mg/mL in gallic acid equivalents) has high scavenging activity, 91.4%, and moreover the phosphomolybdenum assay showed a total antioxidant capacity of the extract linearly increasing with concentration, R^2^ = 0.9927 (Table 2).

### 2.3. Cell Viability and Distribution in the Cell Cycle Phases by Cytofluorimetric Analysis

**Figure 2:**
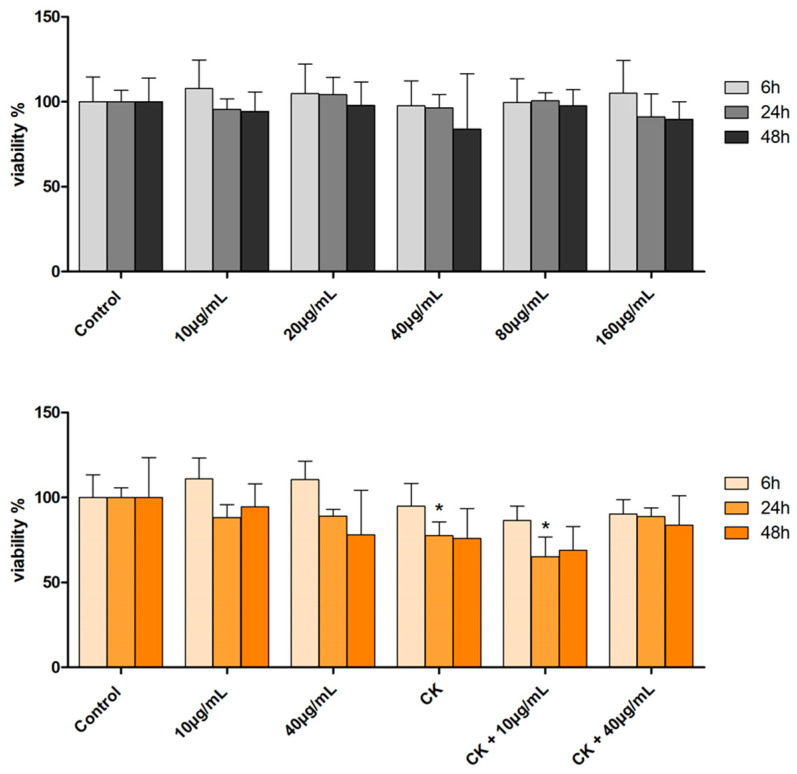
Cell viability by MTT assay. **Top panel**: after treatment with the hydroalcoholic extract of feijoa at 10, 20, 40, 80 and 160 µg/mL for 6, 24 and 48 h. **Bottom panel**: after pretreatment with cytokines for 24 h and subsequent treatment with extract of feijoa at 10 and 40 µg/mL for 6, 24 and 48 h. * indicates statistically significant difference respect to the control at the same time (Student’s *t*-test *p* < 0.05).

Evaluation of the effect on cell viability of feijoa extract at different concentrations (10 to 160 µg/mL) showed that for all samples treated at the three incubation times, the cell viability was comparable to that of the control samples (Figure 2).

When the cells were pre-treated with the proinflammatory cytokines (hereafter CK), TNF-α (Tumour Necrosis Factor alpha) and IL-1β (Interleukin 1 beta) for 24 h and then incubated with feijoa extract at the two different concentrations (10 and 40 μg/mL), chosen on the basis of the results of the previous MTT tests, at 6 h of incubation no change in viability was observed in the extract-treated samples compared to the control, even if the lowest viability values were recorded for all samples pre-treated with cytokines. At 24 h, the cell viability of the samples pre-treated with cytokines only and those with cytokines and extract at a concentration of 10 μg/mL is significantly reduced compared to the control (*p* < 0.05), whereas the viability of the samples with cytokines and feijoa extract 40 μg/mL is comparable with that of the control. At 48 h, the situation is similar to that observed for 24 h, but no change in viability of the treated cells compared to the control is statistically significant (Figure 2).

These data indicate that the extract did not exhibit cytotoxic effects at concentrations up to 160 µg/mL, even after 48 h of incubation, and that at 40 µg/mL it was able to counterbalance the effect on the viability by the cytokines, suggesting therefore a potential cytoprotective activity of the extract in inflammatory intestinal environments.

These findings align with the existing literature on plant-derived compounds with antioxidant and anti-inflammatory properties [36,41,42,43,44].

**Figure 3:**
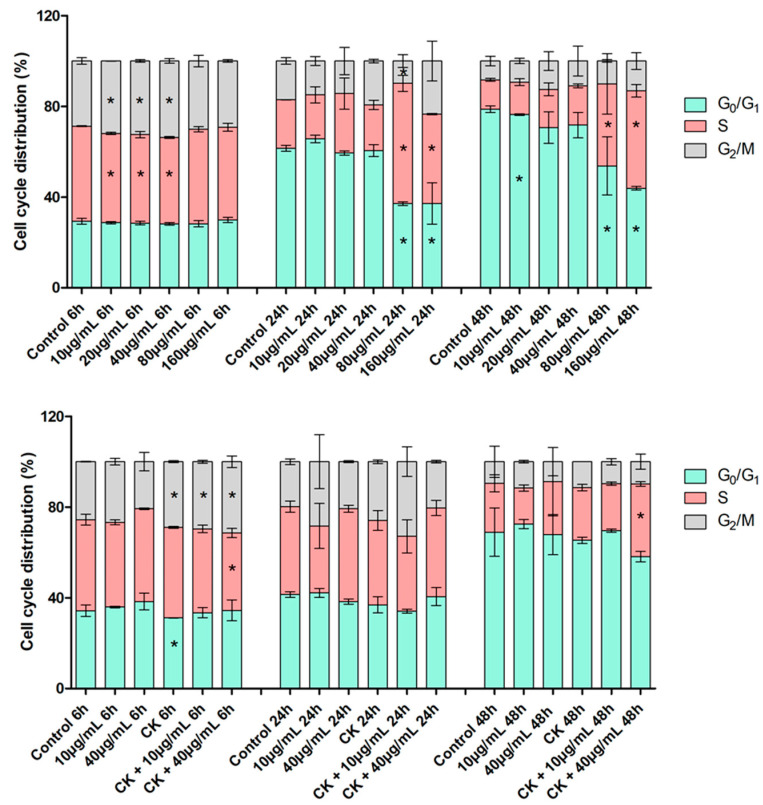
Percentage distribution in the different phases of cell cycle. **Top panel**: after treatment with the hydroalcoholic extract of feijoa at 10, 20, 40, 80 and 160 µg/mL for 6, 24 and 48 h. **Bottom panel**: after pretreatment with cytokines for 24 h and subsequent treatment with extract of feijoa at 10 and 40 µg/mL for 6, 24 and 48 h. * indicates statistically significant difference respect to the control at the same time (Student’s *t*-test *p* < 0.05).

The cytofluorimetric analysis of the distribution of HT29 cells in the different phases of the cell cycle was performed for samples treated with feijoa extract concentrations from 10 to 160 µg/mL for 6, 24 and 48 h as for the viability assays (Figure 3).

For 6 h incubation, a slight decrease in the percentage of cells in S-phase was observed with a subsequent increase in cells in G2/M phase for extract concentrations between 10 and 40 µg/mL (*p* < 0.05); however, it must be said that the proliferative fraction, given by the sum of the percentages of cells in S and G2/M phases, in all treated samples is comparable to that of the control.

At 24 h incubation, a statistically significant increase in the percentage of S-phase cells is observed for samples incubated with the two highest concentrations (80 and 160 µg/mL) and a relative decrease in the number of G0/G1 phase cells (*p* < 0.05).

At 48 h, a similar situation to that at 24 h is observed with an increase for both control and treated samples, in the percentage of cells in G0/G1 phase compared to 6 h and 24 h (*p* < 0.05).

Evaluation of cell cycle phase distribution was subsequently carried out for samples pre-treated for 24 h with 10 ng/mL of proinflammatory cytokines and then incubated with the two concentrations 10 and 40 µg/mL of feijoa extract for 6, 24 and 48 h (as for the viability test) (Figure 3).

At 6 h, for the cytokine-only treated sample, there was a statistically significant increase compared to the control in the percentage of cells in G2/M with a corresponding decrease in that in G0/G1 (*p* < 0.05). For samples pre-treated with cytokines and then incubated with both extract concentrations, there was an increase in respect of the control in the percentage of cells in G2/M and a decrease in that in S-phase, although not all of these differences were statistically significant.

At 24 h of incubation, no statistically significant differences in the percentages of cells in the different phases of the cell cycle were observed in the treated samples compared to the control. Even at 48 h, the distribution of cells in the cycle phases does not differ significantly from the control for all treated samples, except for the S phase of the sample with cytokines and 40 µg/mL extract.

### 2.4. Scratch Assay

**Figure 4:**
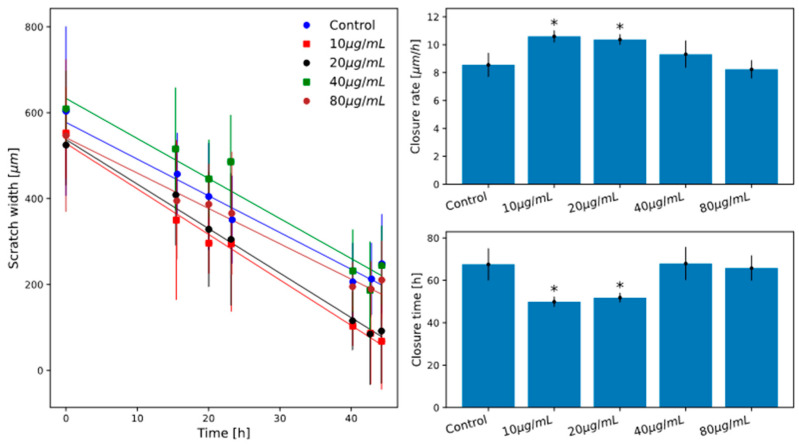
Scratch assay results for HT29 samples treated with 10 µg/mL to 80 µg/mL of feijoa extract. **Left panel**: Scratch closure curves and best-fit linear models (coloured circles and continuous lines as indicated in legend). Error bars represent the standard deviation of experiment triplicates. **Upper right panel**: average scratch closing velocity with standard deviation error. **Bottom right panel**: total scratch closure time with standard deviation error. * indicates statistically significant difference respect to control (Student’s *t*-test *p* < 0.05).

**Figure 5:**
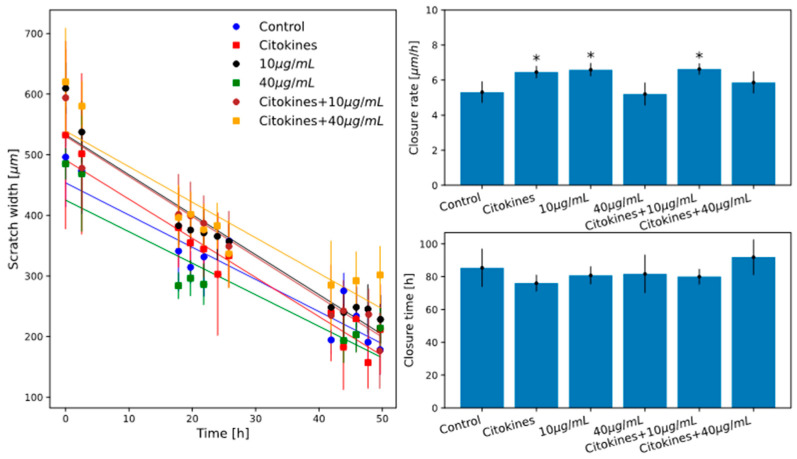
Scratch assay results for HT29 cells treated with 10 µg/mL and 40 µg/mL of feijoa extract and pretreated or not with cytokines. **Left panel**: Scratch closure curves and best-fit linear models (coloured circles and continuous lines as indicated in legend). Error bars represent the standard deviation of experiment triplicates. **Upper right panel**: average scratch closing velocity with standard deviation error. **Bottom right panel**: total scratch closure time with standard deviation error. * indicates statistically significant difference respect to control (Student’s *t*-test *p* < 0.05). As observed in the upper right panel of Figure 4 while for 40 and 80 µg/mL of extract the scratch closure rate is statistically consistent with that of the control, for the 10 and 20 µg/mL concentrations the scratch has a significantly higher closure rate.

In Figure 4 and Figure 5, the measured scratch closure curves for the investigated samples are shown together with the best-fit linear models results.

In a first experiment, we investigated scratch closure for HT29 cells cultured after treatment with feijoa extract at the concentrations of 10, 20, 40 and 80 µg/mL. In Figure 4, the results of the linear model for the above-mentioned samples are shown.

A subsequent experiment was then performed on cells treated with 10 and 40 µg/mL of the extract, both pretreated or not for 24 h with proinflammatory cytokines TNF-α and IL-1β as indicated in the Materials and Methods section. These two extract concentrations were chosen based on the scratch assay results described above and on the cell viability assay results previously presented (they are in fact the lowest and an intermediate concentration of the ones investigated at which treatment shows a significant effect). In Figure 5, the results are shown.

This indicates that the two lower extract concentrations have the effect of stimulating cell migration and proliferation. For the two higher concentrations tested, however, this effect is not observed, and further experiments will be needed to investigate this effect more thoroughly.

The cytokine-pretreated sample (Figure 5) is also characterized by a significantly lower scratch closure rate than the control. The combined treatment with cytokines and 10 µg/mL extract is equally characterized by a significantly higher closure rate than the control and is also consistent with that for treatment with 10 µg/mL extract alone.

Treatment with cytokines and 40 µg/mL extract is characterized by a closure rate statistically comparable with the control and slightly lower (but not significantly) than that of treatment with only 40 µg/mL extract. This suggests that cytokine pretreatment induces a stimulus for cell proliferation and migration similar to that induced by treatment with 10 µg/mL extract.

In contrast, treatment with cytokines and 10 µg/mL extract is not able to further increase the effect of stimulating proliferation and migration because the closure rate remains comparable with that of treatment with only 10 µg/mL extract.

In the case of combined treatment with cytokine and 40 µg/mL extract, the addition of cytokine pretreatment has the effect of slightly (though not significantly) stimulating proliferation and migration that feijoa extract 40 µg/mL treatment alone is unable to bring about.

The significant increase in the scratch closure rate observed for the lower concentrations of extract (10 and 20 µg/mL) as well as for the treatment with cytokines has to be discussed together with the observation that these treatments do not produce significant variations in the cell viability and in the cell cycle phase. This could suggest that these treatments could slightly stimulate the cell migration alone, but it is important to remark that the scratch closure rate is due to the coupled effect of cell migration and proliferation, and it is risky to interpret the two effects in a decoupled manner. For this reason, again, further experiments will be needed for a better and more thorough understanding of these effects.

### 2.5. ROS Production Evaluation by DCFH-DA Assay

**Figure 6:**
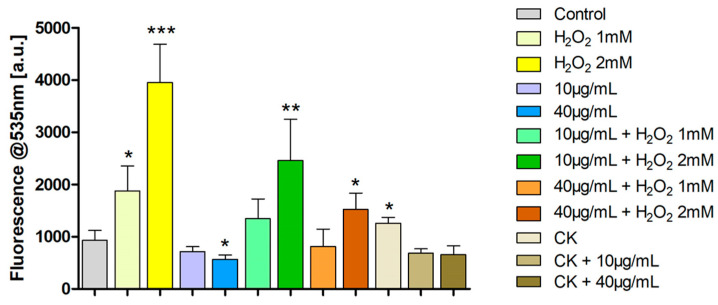
ROS assessment by the DCFH-DA test in HT29 cells treated with the extract of feijoa for 24 h or after pretreatment with cytokines for 24 h or before incubation with two different concentrations of H_2_O_2_, 1 mM or 2 mM for 1 h. *, ** or *** indicates a statistically significant difference with respect of the control (Student’s *t*-test * *p* < 0.05, ** *p* < 0.01, *** *p* < 0.001).

Evaluation of ROS production using the 2′,7′-Dichlorofluorescein diacetate (DCFH-DA) test (Figure 6) shows that treatment with the two chosen concentrations of feijoa extract, 10 and 40 µg/mL, causes a decrease in ROS production compared to control cells; this difference is statistically significant for the 40 μg/mL concentration (*p* < 0.05). Furthermore, treatment with the extract causes a decrease in ROS levels in samples in which oxidative stress was induced with H_2_O_2_ 1 mM and 2 mM (ROS levels increase markedly after exposure to H_2_O_2_ in a dose-dependent manner), so much so that these levels are comparable with those of the control for both concentrations of extract and incubation with H_2_O_2_ 1 mM. Treatment with proinflammatory cytokines also results in a statistically significant increase in ROS compared to the control (*p* < 0.05), although less than that observed with hydrogen peroxide. It is in fact known that an inflammatory state is accompanied by the production of ROS and, therefore, in turn, induces a condition of oxidative stress [45,46].

ROS levels are, on the other hand, comparable with those of the control if, after one day of incubation with cytokines (TNF-α and Il-1β), feijoa extract is added for a further 24 h at a concentration of both 10 μg/mL and 40 μg/mL.

It has to be observed that the 24 h incubation time of the cells with the extract is justified by the results above presented in which this particular incubation time did not produce significant effects in cell viability and distribution in the cell cycle phases.

The results obtained with this test clearly indicate the antioxidant capacity of the extract both when oxidative stress is induced by hydrogen peroxide, one of the most common agents for inducing nonspecific oxidative stress, and when it is due to an inflammatory state. The reduction in intracellular ROS in the presence of hydrogen peroxide occurred in a dose-dependent manner, suggesting a direct scavenging effect of the extract’s bioactive components, likely flavonoids and ellagic acid derivatives [47,48,49,50]. Interestingly, similar results are found also for a wider range of polyphenols such as secoiridoid derivatives [51,52].

### 2.6. Enzymatic Assays, PCA and Pearson Correlation Analyses

**Figure 7:**
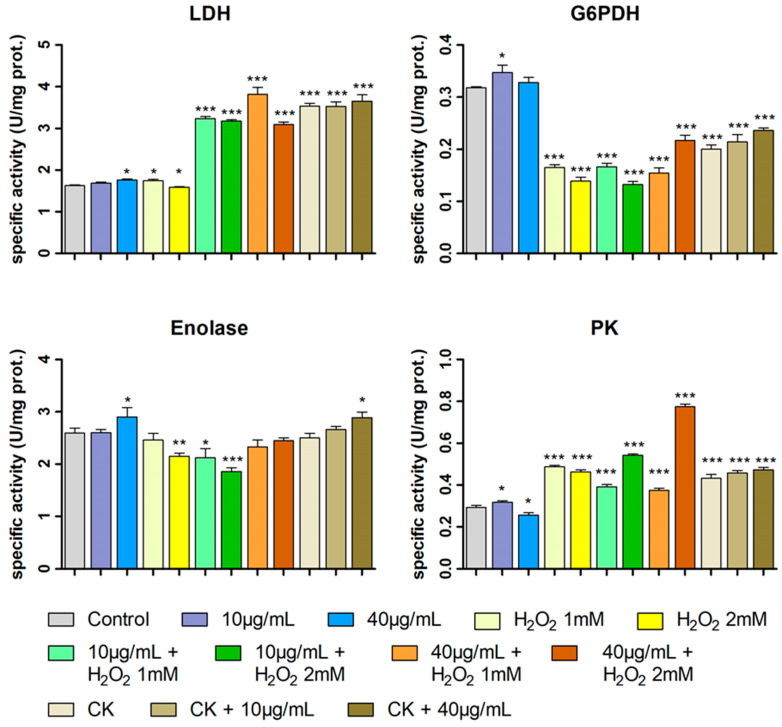
Specific activities of LDH, G6PDH, ENO and PK for control cells, cells treated with H_2_O_2_ 1 mM and 2 mM, 10 µg/mL or 40 µg/mL of the extract of feijoa, cells treated with both the two concentrations of H_2_O_2_ and of the extract, with cytokines and with cytokines and both the concentrations of feijoa extract as specified in the legend. Error bars represent the standard deviation of the three replicates. Samples significantly different from control at the Student’s *t*-test are marked with * (*p* < 0.05), ** (*p* < 0.01) or *** (*p* < 0.001).

The metabolism of HT29 cells subjected to treatments with feijoa extract at concentrations of 10 μg/mL and 40 μg/mL, with H_2_O_2_ 1 mM and 2 mM, with the combinations of extract and H_2_O_2_, with cytokines (TNF-α and IL-1β) and with extract and cytokines (the same treatments as for the DCFH-DA test) was investigated by assessing the specific activity of four metabolic enzymes (Figure 7), two of which are characteristic of the glycolytic pathway (pyruvate kinase, PK and enolase, ENO), one of the phosphate pentoses cycle (Glucose 6-phosphate dehydrogenase, G6PDH) and the other of the pyruvate to lactate reduction pathway (lactate dehydrogenase, LDH). As the link between metabolism and oxidative stress is well known, the purpose of these determinations is not only to study the metabolic effect of feijoa extract, but also its possible role in maintaining the cellular redox state [53,54].

The enzymatic activity variations in the investigated treatments reported in Figure 7 lead to a complex picture, which was further investigated and discussed using also principal component analysis (PCA) and Pearson correlation analyses.

**Figure 8:**
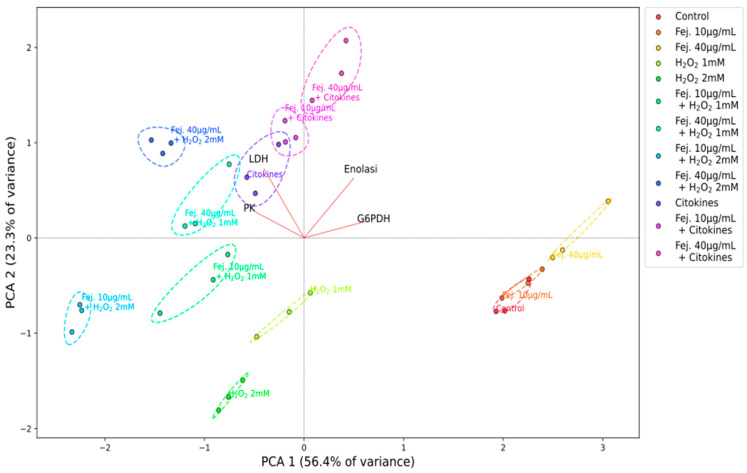
Biplot of the PCA analysis of the enzymatic assays. Coloured circles represent the various samples as specified in the legend. The dashed lines and labels of the same colours help the reader to visualize each cluster. The red lines marked with the enzyme names represent the projections of the relative enzymatic activities on PCA1 and PCA2 coordinates.

PCA analysis allowed observing the overall enzymatic profile of each sample, related to the four assayed enzymes. The results are shown in the biplot of Figure 8. In the figure, three points are shown for each sample that represent the results of the three enzyme assay experiments. The first two principal components PC1 and PC2 account for 79.7% of the variance in the data (PC1 56.4% and PC2 23.3%). Observing Figure 8, it can be noticed that the points for each sample form well-defined clusters that are separated from each other in different regions of the PC1-PC2 plane, revealing that the various samples exhibit clearly distinct enzymatic profiles.

**Figure 9:**
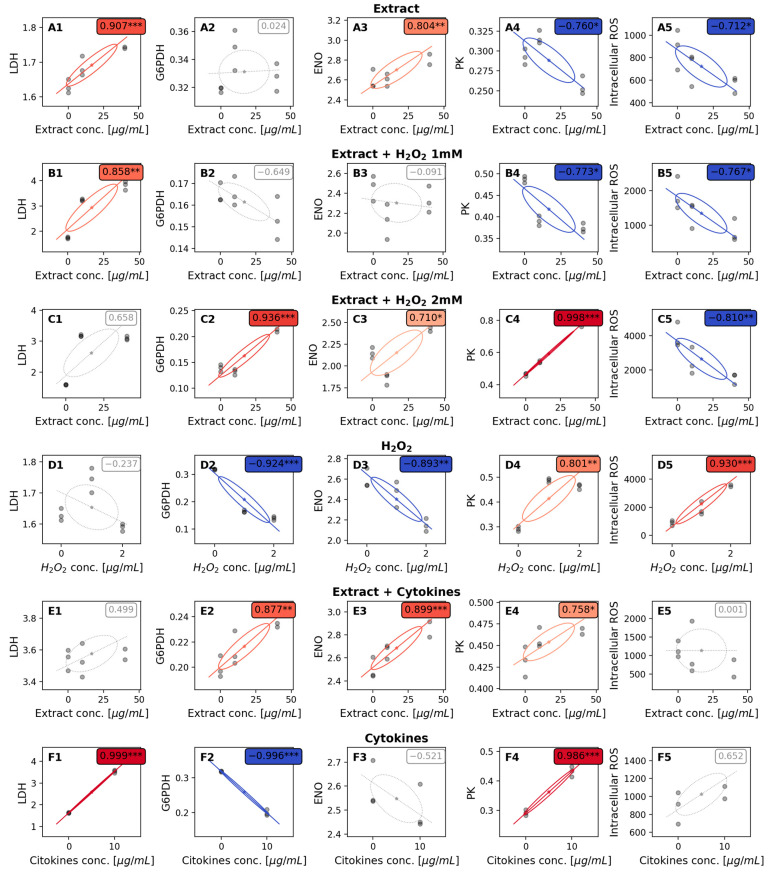
Pearson correlation for the parameters and samples indicated in detail in the text. The grey circles represent the single measurements, and the ellipses and straight lines help to visualize the correlation. The boxes inside each plot contain the Pearson correlation coefficient, * indicates its statistical significance (* *p* < 0.05, ** *p* < 0.01, *** *p* < 0.001). Boxes, ellipses and straight lines are coloured for statistically significative correlations, and the colour scale goes from red for positive correlations to blue for negative ones.

Pearson correlation coefficients (*r*) of the specific activities of LDH, G6PDH, ENO and PK with respect to extract concentration, H_2_O_2_ concentration and cytokine treatment were calculated together with their *p*-value. The intracellular ROS production was also added to this correlation analysis to help discuss the above-mentioned link between metabolism and oxidative stress. The results are shown in Figure 9.

In panels A1–A5 of Figure 9, Pearson correlations for samples treated only with feijoa extract are shown. The *r* values for the significant correlations are indicated in the coloured boxes in each plot (* Pearson *p*-value < 0.05, ** *p*-value <0.01, *** *p*-value < 0.001). The colour scale goes from red for positive correlations to blue for negative ones. Ellipses and lines of the same colour help to visualize the correlation.

In panels B1–B5 and C1–C5, the Pearson correlations for the samples treated with feijoa extract H_2_O_2_ 1 mM and with feijoa extract and H_2_O_2_ 2 mM, respectively, are shown. In panels D1–D5, the correlations for the samples treated only with H_2_O_2_. In panels E1–E5, the correlations for the samples treated with cytokines and extract. In panels F1–F5, Pearson correlations for the samples treated only with cytokines.

Firstly, in Figure 8, it can be observed that clusters related to treatments with feijoa extract alone have different locations from that of the control, indicating that they alter the enzyme profile compared to the control. In particular, a trend in the cluster position with the extract concentration can be observed: where the 10 μg/mL extract cluster is partially overlapped with the control one, the 40 μg/mL extract cluster is more distant, and the cluster position tends to move toward the upper/right quadrant of the biplot.

For the same treatments, from Figure 7, it can be observed that the extract tends to increase the specific activity of all the assayed enzymes; only for PK at the maximum concentration of the extract a decrease in specific activity is observed.

Moreover, from Pearson analysis (panels A1–A5 of Figure 9) can be observed that LDH and ENO specific activities show a significant positive correlation with the extract concentration, compared to the control, while PK activity and ROS intracellular production show a negative one. This last result (panel A5) highlights what is already observed of the effect of reducing intracellular ROS concentration by the extract (Figure 6). In the samples treated only with the extract, it can be assumed that the produced ROS are mainly derived from mitochondrial aerobic activity, involved in the basal metabolism. This negative correlation of intracellular ROS production with extract concentration, taken alone, might suggest a simple chemical scavenger role of the extract against ROS; but, when considered together with the positive correlation with ENO and LDH activity (panels A1 and A3), it might suggest a shift in the cell from an aerobic metabolism, characterised by high mitochondrial activity and ROS production, to an aerobic glycolytic one, characterised by high activity of the glycolysis and lactic fermentation pathway (Warburg effect). Results going in a similar direction have also been found for phytocomplexes rich in polyphenols derived from olive oils and from pâté, a by-product of olive oil production [51,52].

Furthermore, given the negative correlation between PK activity and extract concentration (Figure 9, A4), it could be hypothesised that pyruvate generation is slowing down, resulting in decreased lactate production and NADH oxidation. This would ensure an increased NADH/NAD^+^ ratio with a high reducing power, which would counteract possible oxidative stress.

Observing instead, in Figure 8, the clusters of H_2_O_2_ 1 mM, H_2_O_2_ 2 mM and cytokines, it can be noted that they are shifted to the left of the biplot, compared to the control, but while the first two are shifted downwards, in the 3rd quadrant, the last one is shifted slightly higher, in the 4th quadrant. These shifts with respect to the control cluster seem to indicate that the two treatments change the profile of the tested enzyme activities.

Regarding Pearson correlation analysis for the same treatments (panels D1–D5 and F1–F5 of Figure 9), it can be noted that G6PDH, ENO, PK and intracellular ROS show a similar behaviour for both treatments. This suggest that the two treatments (non-specific oxidative stress, by H_2_O_2_, and induction of the inflammatory state, by cytokines) are very similar except for the positive correlation of LDH activity with cytokine treatment that is not observed with H_2_O_2_ [55].

These two treatments suggest a metabolic picture in which glucose is massively directed towards the glycolytic pathway (PK activity increases), while inducing a significant decrease in the specific activity of G6PDH and, therefore, a lower efficiency of the pentose phosphate pathway.

Regarding the combined treatment with hydrogen peroxide and extract, a trend in the position of the clusters with respect to the concentration of the extract is observed for the samples H_2_O_2_ 1 mM, feijoa extract 10 µg/mL and H_2_O_2_ 1 mM and feijoa extract 40 µg/mL and H_2_O_2_ 1 mM (Figure 8): the cluster shifts to the left with the concentration of 10 µg/mL of the extract and upwards and to the left with the concentration of 40 µg/mL. A similar shift is observed in the clusters of H_2_O_2_ 2 mM, feijoa extract 10 µg/mL and H_2_O_2_ 2 mM and feijoa extract 40 µg/mL and H_2_O_2_ 2 mM.

In more depth, it can be seen from Figure 7 that in samples treated with the extract and then incubated with H_2_O_2_, the effects on the specific activities of the enzymes tested are sometimes different compared to the single treatment with the extract or H_2_O_2_. In some cases, these effects seem to add up and/or amplify suggesting a co-operative mechanism, such as the increase in LDH activity with both the H_2_O_2_ concentration and the higher concentration of extract, as well as the specific activity of PK in the samples treated with the extract and H_2_O_2_ 2 mM. In other cases, the combined treatment seems to show a protective role of the extract with respect to oxidative stress, as in some samples where the activities are different from those of the samples treated with H_2_O_2_ alone and return comparable or tend to those of the control. For example, for ENO in the samples treated with feijoa extract 40 µg/mL and H_2_O_2_ 2 mM, the specific activity is similar to that of the control, while treatment with H_2_O_2_ 2 mM alone shows a significant activity decrease supporting the hypothesis of ROS-induced enzyme damage [56,57]. A similar, but lower recovery of, enzymatic activity is evident also for G6PDH: with extract 40 µg/mL, the pre-treatment specific activity values are closer to the control than in the samples treated with H_2_O_2_ 2 mM alone. On the contrary, a similar behaviour is not found for treatments with H_2_O_2_ 1 mM and extract. As regards the PK activity, the increase in the enzyme activity induced by H_2_O_2_ is decreased by the extract at both its concentrations only for 1 mM of hydrogen peroxide, while for H_2_O_2_ 2 mM, this effect is not present.

In the Pearson analysis of the combined treatments with feijoa extract and H_2_O_2_ (Figure 9, B1–B5, C1–C5), it is the change in the sign of the correlations for a combined treatment compared to the variation in the specific activity due to the corresponding concentration of H_2_O_2_ alone with respect of the control that indicates that the extract counteracts the H_2_O_2_-induced alteration, playing a role of protective agent for the enzyme against an oxidative stress (i.e., in the cases of G6PDH and ENO for the samples treated with H_2_O_2_ 2 mM and extract, panels C2 and C3 of Figure 9).

The observed results regarding the variations in enzymatic activities in combined H_2_O_2_ and extract treatments suggest a framework in which the extract appears to have a protective role by maintaining the cellular redox state more than by inhibiting anaerobic metabolism (Warburg effect). This shift toward an aerobic glycolytic metabolism probably occurs in response to mitochondrial impairment induced by oxidative stress, consistent with what was reported in studies on cells exposed to ROS [58,59]. This protective role also appears to occur through the positive effect on G6PDH activity that, in fact, guarantees an increased supply of NADPH, a coenzyme which contributes to maintaining the redox cellular state through the glutathione reduction pathway [47,48,49,50,60,61,62]. For what concerns the samples related to cytokine treatment (cytokines, feijoa extract 10 µg/mL and cytokines and feijoa extract 40 µg/mL and cytokines), the clusters resulting from the PCA analysis in Figure 8 show distinct positions with respect to the other samples discussed above. In addition, a trend is observed in the position of such clusters with the extract concentration: as this increases, the clusters move upwards and to the right.

For these samples, as can be observed in Figure 7 and panels E1–E5 and F1–F5 of Figure 9, alterations in the specific activity of the enzymes LDH, PK and G6PDH with respect to the control can be observed. In particular, LDH and PK (Figure 7 and panels E1, E4, F1, F4 of Figure 9) show a significant increase in their activity, which is slightly amplified by the treatment with the extract, while G6PDH activity (Figure 7 and panels E2 and F2 of Figure 9) shows a significant decrease, which, however, is slightly compensated for by the treatment with the extract, especially at the highest concentration. This last observation suggests that the extract could also be considered as a protective agent against the effects of cytokines regarding oxidative stress, given the tight relationship between G6PDH activity and the level of reduced glutathione. For what concerns ENO (Figure 7 and panels E3, F3 of Figure 9), incubation with cytokines does not induce significant changes in enzyme activity compared to the control, except in the sample also incubated with 40 μg/mL extract, where this enzymatic activity increases.

The extract’s protective action against oxidative stress on the intracellular production of ROS induced by both concentrations of H_2_O_2_ and by cytokines, evident in Figure 6, is also reported in panels B5, C5 and D5 of Figure 9 for what concerns H_2_O_2_ treatments and in panels E5 and F5 of Figure 9 for what concerns cytokines treatment. Regarding the former treatments (H_2_O_2_ 1 or 2 mM), Pearson analysis shows that ROS production decreases as the extract concentration increases, this holds also for the latter (cytokines) and the related Pearson correlations are not statistically significant (panel F5 of Figure 9), because for both the concentrations of the extract, the intracellular ROS levels are really consistent with that of the control.

In summary, the complex picture emerging from these results indicates a protective role of the extract against oxidative stress conditions and a pro-metabolic effect through the modulation of the activity of important enzymes: an effect compatible with a broad-spectrum nutraceutical action.

However, further in vitro, in vivo and clinical studies regarding the involved biochemical mechanisms are necessary to validate these effects and clarify the safety profile and bioavailability of its active components. In particular, to define the role of the extract from the point of view of its antioxidant properties on HT29 cells, further studies will be crucial on the biochemical systems involved in maintaining the cellular redox state such as the system of the glutathione pathway (activity of the enzymes glutathione reductase, catalase, glutathione peroxidase); the ratio of reduced glutathione/oxidized glutathione; the ratios NADH/NAD+ and NADPH/NADP+; the transcription levels of the PKM2 isoform of PK, particularly involved in situations of oxidative stress and of the genes of the Nrf2 pathway. Furthermore, for clarification, it would be very important to repeat the experiments conducted on HT29 cells with the extract, evaluating the effects exerted by some of the individual molecules that compose it.

## 3. Materials and Methods

### 3.1. Chemicals, Reagents, and Standards

All solvents for HPLC-DAD-MS analyses (HPLC grade), formic acid, ellagic acid and catechin hydrate (analytical grade) were purchased from Sigma Aldrich Chemical Company Inc. (Milwaukee, WI, USA). HPLC-grade water was obtained via distillation and purification with a Labconco Water Pro PS polishing station (Labconco Corporation, Kansas City, MO, USA).

### 3.2. Preparation of Extracts from Feijoa Sellowiana Fruit Pulp

For polyphenol extraction, 15.0 g of freeze-dried pulp was grinded in a mortar and extracted in 100.0 mL of a 70:30 mixture of ethanol and water acidified to pH of 3.2 by formic acid addition, under mechanical stirring, at room temperature for 24 h. The extract was filtered and centrifuged at 14,000 rpm to eliminate solid matrix residues, then rinsed to an exact volume of 100.0 mL with the extraction solvent.

For the biological tests, 60.0 mL of the hydro-alcoholic extract was dried by evaporation under low pressure and rinsed with 5.0 mL of dimethyl sulphoxide (DMSO), to obtain a polyphenolic concentration equal to 20.88 mg/mL GAE according to the Folin–Ciocalteu assay. Suitable dilutions for the different tests were obtained from the concentrated extract.

### 3.3. Evaluation of Total Phenolic Content by Folin–Ciocalteu Assay

The spectrophotometric assay with Folin–Ciocalteu [63] reagent was performed by adding 125 µL of extract appropriately diluted to 500 µL of water and 125 µL of reagent; after 6 min of incubation, 1.25 mL of saturated Na_2_CO_3_ solution and 1.00 mL of water were added and the solution was incubated in the dark for 85 min. The samples were centrifuged for 5 min at 5000 rpm, and the absorbance was measured at 725 nm. The phenol content was determined using a 5-point calibration curve in gallic acid and expressed in gallic acid equivalents (GAEs).

### 3.4. HPLC-DAD-MS Analysis

HPLC-DAD-MS analysis was performed using an HP-1260 liquid chromatograph equipped with a DAD detector and an MSD API-electrospray mass spectrometer (Agilent Technologies, Waldbronn, Germany) operating in positive and negative ionization mode, with a Poroshell EC C18 150 × 3.0 mm, 4 μm column (Agilent Technologies, Waldbronn, Germany) thermostated at 26 °C. The eluents were water at pH 3.2 by addition of formic acid (A) and acetonitrile (B). A multi-step linear gradient was applied starting from 100% A to 100% B, at a flow rate of 0.7 mL/min for a total time of 72 min. The identification of individual compounds was carried out according to retention times, spectrophotometric and spectrometric data, by comparison with the literature data and specific standards where available. Quantification was performed in HPLC-DAD using 5-point regression curves with R^2^ > 0.9998, at the wavelengths of maximum UV-Vis absorbance, applying the correction of molecular weights. Ellagic acid derivatives were calibrated with ellagic acid at 254 nm; procyanidins were calibrated at 280 nm with catechin hydrate. The analyses were performed in triplicate obtaining standard deviations lower than 5%.

### 3.5. Determination of Scavenging Activity by DPPH Test

The DPPH test, is a widely adopted assay to evaluate the scavenging properties of molecules and extracts and involves the use of free radicals to test the ability of substances to act as hydrogen donors or scavengers. DPPH is a stable (solid-state) free radical whose colour changes from purple to yellow when reduced in a scavenging reaction, resulting in a decrease in absorbance of the DPPH solution at 517 nm [64].

The decrease in absorbance is indicative of the scavenging activity of the compounds or extracts tested. For this test, a total of 12 milligrams of DPPH was dissolved in 50 mL of methanol to prepare the stock solution, which was then filtered and diluted prior to use with methanol so that it had an absorbance at 517 nm of about 1. Subsequently, 50 µL of the feijoa extract with concentration 20.88 mg/mL was added to 1.5 mL of the diluted DPPH solution. The sample, prepared in triplicate, was shielded with tin foil and kept in the dark for 30 min before absorbance reading at 517 nm using a plate reader (Infinite M200PRO, Tecan. The percentage scavenging activity was then calculated from the absorbance of the control with DPPH alone (Abs_DPPH) and that of the sample treated with DPPH and extract (Abs_DPPH+Fej) using the formula:

Scavenging Activity % = 100 × (Abs_DPPH − Abs_DPPH + Fej)/Abs_DPPH (1)

### 3.6. Determination of Total Antioxidant Capacity by Phosphomolybdenum Assay

The total antioxidant activity of feijoa extract was evaluated using the phosphomolybdenum assay [65,66]. The analysis is based on the reduction of Mo(VI) to Mo(V) by the sample analysed and the subsequent formation of a green phosphate/Mo(V) complex at acidic pH. The extract at different concentrations (5 µg/mL, 10 µg/mL, 20 µg/mL and 40 µg/mL) was mixed with a solution consisting of 1 mL of 0.6 M sulphuric acid, 28 mM sodium phosphate and 4 mM ammonium molybdate. The tubes containing the reactive solution were then incubated at 95 °C for 90 min. Subsequently, the absorbance of the samples was measured at 695 nm. Then, the concentration of Trolox, a vitamin E analogue, with an equivalent total antioxidant capacity was evaluated by a calibration curve in the range 0.01–1 mM.

### 3.7. HT29 Cell Line

HT29 is an intestinal cell line with epithelial morphology that was isolated in 1964 from a primary tumour obtained from a 44-year-old, Caucasian, female patient with colorectal adenocarcinoma [32,67]. HT29 cells, obtained from the American Type Culture Collection (Manassas, VA, USA), were cultured at 37 °C in a 5% CO_2_ atmosphere in McCoy’s 5A medium with 10% foetal bovine serum (PAN-Biotech, Aidenbach, Germany). Cells were propagated every 2 days by incubation for ~2 min with a 0.25% trypsin/EDTA solution (Merck, Darmstadt, Germany). Cultures were periodically tested for *Mycoplasma* spp. contamination.

### 3.8. Assessment of Cell Viability by MTT Test

The viability of HT29 cells after incubation with feijoa extract was assessed by MTT test; 12,000 cells/well were seeded in a 96-multiwell plate and, after 2 days, were incubated for 6, 24 and 48 h with different concentrations (10, 20, 40, 80 and 160 µg/mL according to total polyphenol content evaluated by in vitro test with Folin–Ciocalteu reagent) of the extract diluted in culture medium. After incubation, cell viability was measured by incubating with 1 mM thiazolyl blue tetrazolium bromide (MERCK, Darmstadt, Germany) in culture medium for 40 min in the dark. Then, the medium was removed and DMSO was added to dissolve formazan crystals. The absorbance signal at 570 nm was read on the multiplate reader (Infinite M200PRO, Tecan, Mannedorf, Switzerland) and the normalized absorbance values were obtained by subtracting the background absorbance at 630 nm was subtracted from signal absorbance. Determinations were also carried out in cells pretreated for 24 h with cytokines (TNF-α and IL-1β 10 ng/mL) to induce an inflammatory state [68,69] and then incubated also with feijoa extract 10 and 40 µg/mL. These extract concentrations were chosen on the basis of the results of MTT tests carried out without cytokine incubation.

### 3.9. Cell Distribution in the Cell Cycle Phases by Cytofluorimetric Analysis

The distribution of the HT29 cells in the different phases of the cell cycle after incubation with feijoa extract was analysed after incubation with the same concentrations of the extract and for the same incubation times of MTT test, also in cells pretreated for 24 h with cytokines to induce inflammation. For these determinations, 250,000 cells were seeded in 6 cm Petri dishes and, after 48 h, incubated with the extracts. At the end of incubation, the cells were collected by trypsinization and were stained with propidium iodide according to the method of Vindeløv [70]. The analysis of the samples was carried out using an FACScan flow cytometer (Becton Dickinson, Milan, Italy). Percentage of cells in the different cycle phases were quantified by ModFit LT software, version 3.0 (Verity Software House Inc., Topsham, ME, USA).

### 3.10. Scratch Assay

HT29 cells were seeded (2.5 × 10^5^) on 6 cm Petri dishes and cultured until ~80% of confluence. The monolayer was then gently scratched with a 200 μL pipette tip thus producing a ~600–1000 μm wide cut. Culture medium and detached cells were removed, and monolayers were washed twice with phosphate-buffered solution (PBS). Fresh complete medium was added to control samples whereas fresh medium with 10 μg/mL and 40 μg/mL of the extract was added to treated samples. Some samples were pretreated for 24 h with cytokines (TNF-α and IL-1β 10 ng/mL) for 24 h before scratching them (the same concentration of cytokines was readded in the medium after washing samples). Samples were then observed at the microscope as detailed in the following: this moment is considered as 0 delay time of the scratch closure to which we refer in the following. Experiments were performed in triplicate.

Scratch assay imaging was performed with an inverted microscope in phase contrast configuration (Leica DM IL, Leica Microsystems GmbH, Wetzlar, Germany) equipped with a 5x objective and a VisiCam 5 Plus camera (2560 × 1922, ~2.2 × 2.2 μm pixels, Avantor Inc., Radnor Township, Pennsylvania), obtaining a field of view of ~1.5 × 1.5 mm necessary to observe the entire scratch width (~0.6–1.0 mm). Images of the scratch were acquired approximately every 2 h (except for the unavoidable night-time gap) from the starting time to ~25–40 h, thus guaranteeing 10–12 images.

Dedicated image analysis routines written in the Python language (Python Software Foundation, version 3.8, available at https://www.python.org/) allowed measurement of the scratch width for each image. Then, for each sample and delay time, mean scratch width and standard deviation on the triplicate was calculated. Finally, the scratch width vs. delay time curve for each sample was constructed.

This curve was then modelled by means of least squares minimization with a linear model calculating the scratch closure velocity and closure-time (i.e., the time at which the scratch is completely closed according to the linear model). For any detail regarding image analysis and scratch closure modelling, refer to [71].

### 3.11. Evaluation of Intracellular ROS Production

Intracellular ROS production was measured by means of an assay with 2′,7′-dichlorofluorescein diacetate (DCFH-DA, Merck, Darmstadt, Germany), a molecule that can freely cross cell membranes. DCFH-DA is enzymatically hydrolysed by intracellular esterases to non-fluorescent dichlorofluorescein (DCFH), thus losing its ability to backscatter across the membrane. In the presence of ROS, DCFH is oxidised to highly fluorescent DCF, which can be detected and quantified [72]. For this test, cells were plated in a 96-multiwell plate at a concentration of 12,000 cells per well. The next day, some samples were pre-treated with TNF-α and IL-1β 10 ng/mL. After 24 h, the cells were incubated with the extract at the chosen concentration of 10 and 40 µg/mL with the addition of 25 μM DCFH-DA. Finally, after a further 24 h, designated samples were exposed to H_2_O_2_, at concentrations of 1 or 2 mM, for one hour. These H_2_O_2_ concentrations were selected as they induce detectable ROS production in HT29 cells. After H_2_O_2_ treatment, all samples were washed twice with PBS and the relative levels of fluorescence emission were quantified using a multi-well plate reader (with excitation at 485 nm and emission at 535 nm).

### 3.12. Enzymatic Assays

For the determination of the activity of some key enzymes of the main metabolic pathways, 250,000 cells were plated in Petri dishes (ø 10 cm). After 24 h, some samples were pre-treated with the two cytokines (10 ng/mL). The next day, the cells were incubated with the extract at the concentration of 10 and 40 µg/mL and after 24 h some of the samples incubated with only the two extract concentrations were treated for 1 h with H_2_O_2_ 1 mM or 2 mM. At the end of this latest treatment, the cells of all samples were collected by trypsinization. Cells were quickly rinsed in ice-cold PBS and frozen at −80 °C.

At the time of use, after thawing, cells were lysed by sonication (three short bursts) at 4 °C in 50 mM Tris, pH 7.4, containing 3 mM dithiothreitol and Sigma protease inhibitors mix (1/100, *v*/*v*) and centrifuged at 12,000 *g* in a microcentrifuge at 4 °C for 30 min. The supernatants were used for the evaluation of enzyme activities with respect to protein content. The total protein content was determined spectrophotometrically according to the Bradford method [73], using a reagent by Sigma-Aldrich.

The determinations of the enzymatic activities of enolase (ENO, EC 4.2.1.11), pyruvate kinase (PK, EC 2.7.1.40), lactate dehydrogenase (LDH, EC 1.1.1.27) and glucose-6-P-dehydrogenase (G6 PDH, EC 1.1.1.49), were performed at 37 °C, according to Bergmeyer [74], with slight modifications, continuously following NADH or NADPH appearance/disappearance at 340 nm, using a UV-2100 spectrophotometer (Shimadzu, Columbia, MD, USA). All the enzymatic reactions were started by adding the substrate. One unit of activity is defined as the quantity of enzyme which transforms 1 µmol of substrate in 1 min. The value of 6.22 mM^−1^ cm^−1^ is considered the NADH (or NADPH) molar extinction coefficient. All the reagents used for enzymatic assays were purchased from Merck (Darmstadt, Germany).

### 3.13. Principal Component Analysis (PCA) of Enzyme Activities

To discuss how the investigated treatments influence the overall profile of the four enzyme activities measured for the cells, PCA analysis was performed [75]. PCA is a dimensionality reduction technique commonly adopted in data clustering problems. This technique finds the coordinate transformation that maximises the variance in the data along the new coordinates (called principal components, PCs). The clustering of data points is then investigated by considering only the PCs that account for the highest fraction of the variance, thus reducing the dimensionality of the data. In each of the three enzymatic assay experiments, triplicate samples were prepared as follows: control, H_2_O_2_ 1 mM, H_2_O_2_ 2 mM, feijoa extract 10 μg/mL; feijoa extract 40 μg/mL, H_2_O_2_ 1 mM with feijoa 10 μg/mL, H_2_O_2_ 2 mM with feijoa 40 μg/mL, cytokines, cytokines with feijoa 10 μg/mL and cytokines with feijoa 40 μg/mL. Each point in the PCA data represents the average of that sample over one experiment. The principal components for this data set were calculated and the results represented in the form of a biplot showing the two principal components accounting for the highest fraction of the variance in the original data. The PCA analysis was performed separately for the two feijoa concentrations: as the decomposition technique is based on the variance in the data, including the two feijoa concentrations in a single dataset could increase the variance in the data and lead to misinterpretation of the results. Therefore, PCA was performed independently on one dataset relating to the 10 μg/mL concentration of feijoa extract and on another dataset relating to the 40 μg/mL dose of feijoa extract.

### 3.14. Statistical Analyses

All experiments were performed in triplicate. The differences between treated and control samples observed in the biological activity assessment tests, such as the MTT test, cytofluorimetric analysis, ROS production test and enzyme activity, were analysed using Student’s *t*-test (significance level of 0.05).

Furthermore, to assess the statistical significance of the time course of the MTT test and enzyme activity tests, a two-way analysis of variance (ANOVA test) interaction parameter with significance level *p* < 0.05 was performed.

ANOVA and Student’s *t*-tests were performed using GraphPad Prism version 5.03 (GraphPad Software, San Diego, CA, USA, www.graphpad.com).

Pearson correlation analysis of enzymatic activities and ROS production with treatments was carried out according to [76,77].

## 4. Conclusions

Feijoa fruit showed interesting nutritional and biological properties, even though few studies are available concerning its content of bioactive compounds. In this study, the quali-quantitative analysis of polyphenolic fraction indicated the presence of an interesting content and a large variety of polyphenols mainly belonging to the subclasses of hydrolyzable and condensed tannins.

Feijoa hydroalcoholic extract resulted not cytotoxic on HT29 intestinal epithelial cells at the investigated concentrations and exerted a significant protective effect cells subjected to oxidative (H_2_O_2_) and inflammatory (cytokine-induced) stresses as shown by the experiments on intracellular ROS combined with a pro-metabolic effect through the modulation of the activity of important enzymes (LDH, G6PDH, ENO, PK).

From the results here presented, feijoa extract appears to be a promising candidate for the development of preventive or adjuvant nutraceutical strategies in the treatment of inflammatory and oxidative intestinal conditions, such as ulcerative colitis or irritable bowel syndrome. Dietary supplementation with feijoa extracts may represent a promising frontier in the field of translational gastrointestinal medicine.

Future studies will be aimed at clarifying the biochemical mechanisms involved in the antioxidant and pro-metabolic effects shown by the extract, also evaluating the role of the individual molecules that compose this phytocomplex.

Moreover, many of the other activities of the wide-ranging research on *Feijoa sellowiana*, in particular, agronomic and genetic, are still ongoing and the results will be reported in further publications.

## Figures and Tables

**Table 1:** Quali-quantitative HPLC-DAD-MS analysis of feijoa pulp extract. Results in mg individual compounds per gram of freeze-dried (FDP) or fresh (FP) pulp, considering an average water content of 83%. Data are the means of three measurements, with standard deviations < 5%.

		mg/g FDP	mg/g FP
1	Pedunculagin isomer I	0.157	0.027
2	Pedunculagin isomer II	0.245	0.042
3	HHDP-glucose	0.023	0.004
4	Flavonoid derivative	traces	traces
5	Casuarictin/potentillin	0.028	0.005
6	Ellagic acid hexoside I	0.020	0.003
7	Ellagic acid arabinoside I	0.059	0.010
8	Ellagic acid arabinoside II	0.012	0.002
9	Ellagic acid hexoside II	0.017	0.003
10	Ellagic acid derivative *m*/*z* 491	0.120	0.020
11	Ellagic acid derivative *m*/*z* 423	0.024	0.004
12	Ellagic acid derivatives calibrated as ellagic acid	0.109	0.019
13	Procyanidin dimer I	0.147	0.025
14	Procyanidin dimer II	0.337	0.057
15	Procyanidin dimer III	0.079	0.013
16	Unknown compound calibrated as ellagic acid	0.054	0.009
Total polyphenols		1.431	0.243

**Table 2:** Total antioxidant capacity by phosphomolybdenum assay for four concentrations of feijoa extract and the concentration of Trolox with equivalent antioxidant capacity.

Feijoa Extract Concentration (µg/mL GAE)	Total Antioxidant Capacity	Trolox Concentration with Equivalent Antioxidant Capacity (mM)
5 µg/mL	0.899 ± 0.014	0.201 ± 0.004
10 µg/mL	1.396 ± 0.014	0.343 ± 0.004
20 µg/mL	2.18 ± 0.06	0.568 ± 0.017
40 µg/mL	3.295 ± 0.09	0.887 ± 0.026

## Data Availability

Data is contained within the article.

## Abbreviations

The following abbreviations are used in this manuscript:

HHDP-glucose	Hexahydroxydiphenoyl-glucose
DPPH	2,2-diphenyl-1-picrylhydrazyl
PCA	Principal Component Analysis
ROS	Reactive Oxygen Species
GAE	Gallic Acid Equivalent
FDP	Freeze-Dried Pulp
FP	Fresh Pulp
CK	Cytokines
TNF-α	Tumour Necrosis Factor alpha
IL-1β	Interleukin 1 beta
DCFH-DA	2′,7′-Dichlorofluorescein diacetate
PK	Pyruvate Kinase
ENO	Enolase
LDH	Lactic Dehydrogenase
G6PDH	Glucose-6 Phosphate Dehydrogenase
PBS	Phosphate-Buffered Solution
DMSO	Dimethyl sulphoxide
